# Genetic Risk Scores for the Determination of Type 2 Diabetes Mellitus (T2DM) in North India

**DOI:** 10.3390/ijerph20043729

**Published:** 2023-02-20

**Authors:** Lisa Mitsuko Shitomi-Jones, Liz Akam, David Hunter, Puneetpal Singh, Sarabjit Mastana

**Affiliations:** 1 School of Sport, Exercise and Health Sciences, Loughborough University, Epinal Way, Leicestershire, Loughborough LE11 3TU, UK; 2 Department of Human Genetics, Punjabi University, Patiala 147002, India

**Keywords:** India, polygenic risk score, polymorphism, type 2 diabetes

## Abstract

Background: Globally, type 2 diabetes mellitus (T2DM) is one of the fastest-growing noncommunicable multifactorial and polygenic diseases, which leads to many health complications and significant morbidity and mortality. South Asians have a high genetic predisposition to T2DM, with India being home to one in six diabetics. This study investigates the association of selected genetic polymorphisms with T2DM risk and develops a polygenic risk score (PRS). Methods: A case–control study recruited fully consented participants from a population of Jat Sikhs in north India. DNA samples were genotyped for a range of polymorphisms and odds ratios were calculated under several genetic association models. Receiver operating characteristic (ROC) curves were produced for combinations of the PRS and clinical parameters. Results: The GSTT1(rs17856199), GSTM1(rs366631), GSTP1(rs1695), KCNQ1(rs2237892), ACE(rs4646994), and TCF7L2(rs12255372; rs7903146; rs7901695) polymorphisms were associated with increased T2DM risk (*p* ≤ 0.05). No association was observed with IGF2BP2(rs4402960) or PPARG2(rs1801282). The weighted PRS was found to be significantly higher in patients (mean = 15.4, SD = 3.24) than controls (mean = 11.9, SD = 3.06), and t_(454)_ = −12.2 (*p* < 0.001). The ROC curve analysis found the weighted PRS in combination with clinical variables to be the most effective predictor of T2DM (area under the curve = 0.844, 95%CI = 0.0.808–0.879). Conclusions: Several polymorphisms were associated with T2DM risk. PRS based on even a limited number of loci improves the prediction of the disease. This may provide a useful method for determining T2DM susceptibility for clinical and public health applications.

## 1. Introduction

Diabetes is one of the fastest-growing chronic illnesses in the world, with the number of diabetics almost quadrupling since 1980 [1]. The most common type of diabetes is type 2 diabetes mellitus (T2DM), accounting for around 90% of all patients [2]. Risk factors of T2DM include genetic predisposition, as well as lifestyle factors such as obesity and physical inactivity [3,4]. T2DM is characterised by insulin resistance, resulting in poor blood glucose control and hyperglycaemia [5]. This can cause numerous health complications including nerve damage, heart disease, and kidney failure, with an estimated global burden of USD 3.1 trillion [6,7]. 

In 2019, diabetes was the ninth leading cause of mortality globally, with over 1 in 10 adults aged 20–79 suffering from the disease [2,8,9]. Prevalence is projected to rise by around 45% by the year 2045, with the greatest increases expected in low- to middle-income countries [2]. India has the second-largest number of diabetics in the world, home to one in six of all patients [2]. Driven by high genetic predisposition and worsening lifestyle factors, the prevalence of diabetes in India is projected to further increase by 74% by 2045 [2,10]. Undiagnosed or uncontrolled cases of T2DM can lead to both microvascular and macrovascular damage [11]. Development of microvascular disease can result in complications such as impaired vision (retinopathy), kidney damage (nephropathy), nerve damage (neuropathy), and amputation [12]. Cardiovascular disease resulting from macrovascular damage is the leading cause of mortality in diabetics, typically resulting from coronary heart disease, stroke, and peripheral arterial disease [13,14].

The burden of T2DM in India is exacerbated by the low diagnosis rate, with an estimated 57% of diabetics going undiagnosed [2,9]. This allows for many diabetics to go untreated and develop complications, after which most healthcare costs come out of pocket [15]. The combination of high disease prevalence and inadequate healthcare has resulted in T2DM having the highest health burden of noncommunicable diseases in India when measured by disability-adjusted life-years (DALYs) [16].

T2DM is a multifactorial disease, with risk primarily driven by lifestyle factors such as obesity, physical inactivity, and poor diet [3]. Indians have a high predisposition to T2DM, with the disease developing at younger ages and lower body mass index (BMI) values than Western countries [10,17]. Studies have demonstrated that Indians typically have higher bodyfat and increased central adiposity at a given BMI compared to other ethnicities, as well as a greater propensity for dyslipidaemia and insulin resistance [17,18,19]. This increased predisposition for T2DM is also demonstrated by a higher prevalence of diabetes in the Indian diaspora than native populations of those countries [10]. T2DM is also a polygenic disease with a high level of heritability [20]. The genome-wide association studies (GWAS) have identified >150 loci contributing to approximately 10–15% of genetic predisposition, although comprehensive studies in Indian populations are still limited [4,10]. In this study, we analysed 10 known susceptibility loci whose role and pathological functions are implicated in diabetes directly or indirectly. The details of the loci and functions along with some previous studies are listed in Table 1.

The aim of this study is to assess the genetic association of the polymorphisms listed in Table 1 with T2DM risk in a Jat Sikh population in north India. Additionally, this study aims to produce a polygenic risk score (PRS) to capture the cumulative effect of these polymorphisms on T2DM risk. As this is an endogamous population, the ethnic homogeneity may give a novel insight into the association between these genetic variants and risk of developing T2DM. In addition, establishing the combined effect of these polymorphisms could allow for improved screening of individuals with a genetic predisposition for T2DM and may contribute to the development of personalised medicines. 

## 2. Methods

Participants were recruited for this case–control study from the states of Punjab (Patiala, Jalandhar, and Kapurthala Districts) and Haryana (Ambala District) of north India, as detailed by Mastana and colleagues [23]. Patients and controls were recruited from a range of primary health care centres in many villages in the above districts. Primary health care doctors and village heads advertised the study and asked volunteers to participate in the research study. A total of 225 patients (133 males and 92 females) and 231 controls (112 males and 119 females) were enrolled in the study. Participants were 30 to 70 years of age and belonged to the endogamous population of Jat Sikhs, as determined by evaluation of family history. Age, sex, and family history of T2DM were determined via questionnaire, and participants (patients and controls) were matched based on age and sex wherever possible. All participants provided written informed consent and the research protocol received approval from the Loughborough University Ethical Advisory Committee, as well as relevant local hospitals and health authorities. The sample size calculation using software package Quanto version 1.2 [36] suggested that a sample of 215 patients and 215 controls should be sufficient to detect an odds ratio of 1.5 at 80% power using allele frequency information from previous studies. 

T2DM patients were included based on clinical records, medications, and OGTT using criteria established by the American Diabetes Association (2004) [37]. Medical history indicated either a fasting plasma glucose (FPG) level ≥ 7.0 mmol/L or ≥126 mg/dL after a minimum 12 h fast or 2 h post glucose level (oral glucose tolerance test or 2 h OGTT) ≥11.1 mmol/L or ≥200 mg/dL on more than one occasion with symptoms of diabetes. The 2 h OGTTs were performed following WHO criteria (75 gm oral glucose load). Any patients with cardiovascular or kidney ailments were excluded from recruitment.

All controls were unrelated, apparently healthy individuals, free of any diabetic phenotypes and vascular diseases. Controls were matched for sex and age and geographical location with patients, where possible. Controls were also given 2 h OGTT for ruling out the presence of impaired glucose tolerance (IGT), which could lead to pre-diabetes/diabetes. Any controls suffering from any cardiovascular or kidney ailments were excluded from the study/analyses. 

All anthropometric measurements including height, weight, waist and hip circumferences, and blood pressure were measured using standardised procedures. Hypertension was defined by systolic blood pressure ≥ 140 mmHg and diastolic blood pressure ≥ 90 mmHg or taking blood pressure medication. Body mass index (BMI) was calculated as [weight (kg)/height (m)^2^]. All biochemical measurements (total cholesterol (TC), high-density lipoprotein (HDL), triglycerides (TG), and very low-density lipoprotein (VLDL)) were estimated on automated analysers using standard clinical kits. 

Genes/SNPs analysed in this study were selected based on validated genes/SNPs having functional effects on the diabetes phenotype directly or indirectly: having allele frequency at least 5% in different populations and previous association with either type 2 diabetes or intermediate pathways. In this way, 8 genes with 10 SNPs were identified and analysed in this study using PCR- (GSTT1, GSTM1, and ACE), PCR-RFLP- (GSTP1, KCNQ1, and IGF2PB2) and TaqMan-based (PPARG2 and TCF7L2) genotyping. Role and putative pathological function of these loci are included in Table 1 along with suggested associations. DNAs were extracted from whole blood with salting out procedure [38] and using specific primers/TaqMan assays. All genotyping was carried out at Loughborough University without the knowledge of disease status to avoid any bias in genotyping. All PCR/PCR-RFLP gels were independently scored by two independent researchers and 100% consistency in genotyping was observed. Approximately 15% of the samples were re-analysed to confirm the internal consistency in genotyping and replication.

### Statistical Analyses

Anthropometric and clinical parameters were assessed by an independent sample *t*-test to assess differences between the patient and control group. Genotype frequencies, allele frequencies, and Hardy–Weinberg equilibrium (HWE) were determined using an online calculator (https://ihg.helmholtz-muenchen.de/cgi-bin/hw/hwa1.pl, accessed on 1 March 2021). ORs were calculated at each locus under codominant, dominant, recessive, and log-additive association models using the SNPStats web tool, with and without adjustment for age and BMI (https://www.snpstats.net/start.htm, accessed on 10 March 2021) [39]. Haplotypes and associated ORs were computed for the rs12255372, rs7903146, and rs7901695 loci on the *TCF7L2* gene.

The genetic risk scores, also called polygenic risk scores (PRS), were calculated for each participant as the total number of risk alleles from the 10 polymorphisms (presence of the null genotype at *GSTT1* and *GSTM1* was coded as one risk allele per genotype). Weighted PRSs were calculated using the log-additive ORs, or the recessive ORs in the case of *GSTT1* and *GSTM1*, as detailed in the equation below:

$$Weighted\;PRS=\sum \left(n\times OR\right)$$

where *n* = number of risk alleles at a locus; *OR* = odds ratio.

The crude and weighted PRSs were compared via an independent sample *t*-test. Levene’s test was used to assess the homogeneity of variance, and normality of the PRSs were determined by calculation of z-scores of skewness and kurtosis [40]. All statistical analyses were carried out using the Statistical Package of Social Sciences (SPSS) for Windows, version 27 (SPSS, IBM Corp (2020) Armonk, NY, USA).

Binary logistic regression analyses were carried out separately for the genotypes and weighted PRS, with and without the inclusion of clinical covariates. The following demographic and clinical variables were adjusted in regression analyses; sex, age, BMI, waist circumference, hip circumference, bodyfat, SBP, DBP, cholesterol, triglycerides, and high-density lipoprotein. 

Receiver operating characteristic (ROC) curves are a widely used and well-established method of determining the discrimination accuracy of predictive models [41]. In the present study, ROC curves were generated to compare the disease-predictive capacities of the clinical variables, crude and weighted PRSs.

## 3. Results

### 3.1. Descriptive Statistics

Patients were significantly older (*p* < 0.01) and had larger waist circumferences and WHRs (*p* < 0.01), as well as higher systolic blood pressure (SBP) and concentrations of triglycerides (TG), low-density lipoprotein (LDL), very low-density lipoprotein (VLDL) (*p* < 0.05), and lower high-density lipoprotein (HDL) than controls (*p* = 0.009) (see Supplementary Table S1). Results of analysed genetic loci are presented in Table 2; only the *PPARG2* locus deviated from the HWE in the patient group (*p* = 0.006).

### 3.2. Odds Ratios

Genotype ORs are presented in Table 3. The null genotypes at both *GSTT1* and *GSTM1* were found to significantly increase the risk of T2DM (OR = 2.16, 95%CI = 1.39–3.36 and OR = 2.81, 95%CI = 1.91–4.13, respectively). The V allele of *GSTP1* and C allele of *KCNQ1* were both found to be associated with T2DM risk, with the greatest significant risk associated with the V/V genotype under the codominant model for *GSTP1* (OR = 3.10, 95%CI = 1.67–5.76) and the C/C genotype under the recessive model for *KCNQ1* (OR = 1.67, 95%CI = 1.13–2.47). No significant associations were found for either *IGF2BP2* or *PPARG2* under any of the association models (*p* ≥ 0.05).

TD2M risk was associated with the deletion polymorphism of *ACE* under all models, with a log-additive OR of 2.06 (95%CI = 1.55–2.72) and co-dominant OR for the D/D genotype of 4.11 (95%CI = 2.33–7.25). The rs12255372, rs7903146, and rs7901695 loci of *TCF7L2* were all significantly associated with T2DM under the codominant and log-additive models, with log-additive ORs of 2.06 (95%CI = 1.45–2.93), 1.43 (95%CI = 1.03–1.97), and 2.08 (95%CI = 1.56–2.77), respectively. The rs12255372 and rs7901695 retained significant association under the dominant model, whilst rs7903146 and rs7901695 were significant under the recessive model (*p* < 0.05).

Haplotype analyses of the *TCF7L2* loci are presented in Table 4. A relatively low level of linkage disequilibrium was observed between rs12255372 and rs7903146 (D′ = 0.089; *p* = 0.015). No significant associations were found for other combinations. Eight haplotypes were observed using TCF7L2 SNPs(rs12255372, rs7903146, and rs7901695). GCT was the most common haplotype and was considered as a reference. Five haplotypes (GCC, GTT, TCT, TCC, and GTC) were found to increase the risk of T2DM when compared to the reference haplotype (*p* < 0.05) and all ORs remained significant after adjustment for age and BMI.

### 3.3. Polygenic Risk Score and Receiver Operating Characteristic Curves

**Figure 1:**
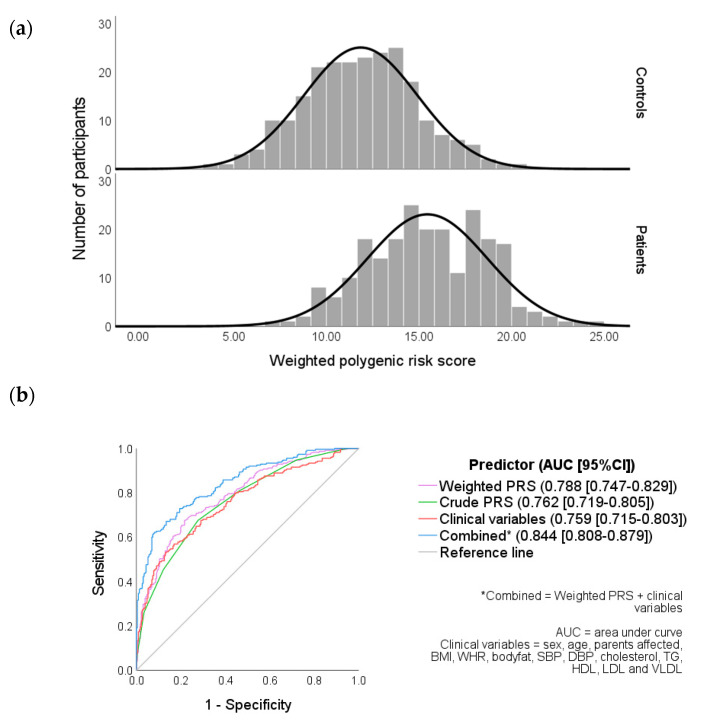
(**a**) Weighted PRS distribution in controls and patients and (**b**) receiver operating characteristic (ROC) curves of clinical variables, weighted and crude PRS.

The distributions of weighted PRS in controls and patients are illustrated in Figure 1a. Both were found to be normally distributed at an α = 0.05 probability level (controls: Z_skew_ = 0.456 and Z_kurtosis_ = −0.386; patients: Z_skew_ = −0.161 and Z_kurtosis_ = −1.139). An independent sample *t*-test revealed that the weighted PRS for patients (mean = 15.4, SD = 3.24, and *n* = 225) was significantly different from controls (mean = 11.9, SD = 3.06, and *n* = 231), and t_(454)_ = −12.2 (*p* < 0.001).

The combined (weighted PRS and clinical variables together) area under the curve (AUC) was a significantly better predictor (0.84) of the diabetes risk compared to clinical variables (0.76) or weighted PRS (0.79) individually. 

### 3.4. Binary Logistic Regression Analyses 

The binary logistic regression analysis of genetic loci (Supplementary Table S2) revealed largely similar associations as observed in standard genetic analyses (Table 3). After inclusion of selected clinical and demographic variables, significant associations remained for genotypes on the *GSTM1*, *GSTP1*, *KCNQ1*, *ACE*, and *TCF7L2* genes (Table S2) (*p* < 0.05). Having a parent affected by T2DM was shown to be an independent predictor of T2DM, with both parents affected increasing the risk of an individual by 9.3 times (95%CI = 2.26–38.1). Increased SBP was also shown to be a predictor of T2DM, while greater hip circumference and cholesterol appeared to provide minor protective effects (Exp(β) = 1.02, 95%CI = 1.006–1.035; Exp(β) = 0.994, 95%CI = 0.988–0.999, respectively). LDL and VLDL were excluded from binary logistic regression models due to collinearity.

The effects of clinical variables remained relatively consistent in the binary logistic regression model for the weighted PRS (Table S3). A one-unit increase in weighted PRS was associated with 1.4 times increased odds of T2DM (95%CI = 1.32–1.54), which remained consistent after inclusion of clinical parameters into the model (*p* < 0.05). 

## 4. Discussion

This is the first known study evaluating the combined effects of 10 polymorphisms and the resultant genetic risk score on T2DM in a Jat Sikh population from north India. The *GSTT1*, *GSTM1*, *GSTP1*, *KCNQ1*, *ACE*, and *TCF7L2* polymorphisms were found to significantly increase T2DM risk, whereas no association was observed for *IGF2BP2* or *PPARG2*. The PRS was found to be an independent predictor of T2DM, with increased predictive capacity when combined with clinical variables.

### 4.1. Clinical Parameters

On average, patients were found to be older than controls (Table S1). However, all ORs were significant after adjusting for age and BMI, indicating that the increased risks associated with the polymorphisms were not due to increased T2DM risk with age (Table 3 and Table S2). Patients also had larger waist circumferences and WHR, despite no significant difference in BMI, which suggests WHR is a better predictor of T2DM than BMI [42]. Patients had higher levels of TG, LDL, and VLDL and lower levels of HDL, which is characteristic of dyslipidaemia commonly found in diabetics due to the influence of insulin resistance on lipid metabolism [43,44,45].

### 4.2. Genetic Associations

All loci were found to be in HWE for controls, granting confidence that the sample can be regarded as representative of the general population (Table 2).

The null genotypes of the *GSTT1* and *GSTM1* loci were associated with an increased risk of T2DM (Table 3), which is consistent with previous studies on Asian populations [21,23]. As GST enzymes catalyse detoxification reactions, it is conjectured that the null polymorphisms of these loci are linked to lowered enzyme activity, therefore resulting in increased macromolecular damage from reactive oxygen species [46,47]. Increased oxidative stress has been demonstrated to impair glucose metabolism, and thus T2DM pathogenesis [48,49]. The *GSTP1 V* allele was also found to significantly increase T2DM risk, which is supported by previous studies [23,47] that observed low GST activity in participants with the V/V genotype. Some of the variation in the effects of the GST polymorphisms on T2DM risk may be attributed to genetic interactions with environmental exposures. India is Asia’s leading producer of pesticides and ranks twelfth in the world for usage [50]. Pesticide usage has been shown to promote oxidative stress, with research suggesting that exposure may compound the deleterious effects of GST polymorphisms [51,52]. The present study was conducted in a population of Jat Sikhs, who are traditionally agriculturists and are the predominant landowners of Punjab [53]. These environmental interactions may have influenced the findings of the present study and partly explain the higher T2DM risk observed with GST polymorphisms in comparison to previous studies.

*KCNQ1* codes for the alpha subunit of a family of voltage-gated potassium channels thought to regulate the secretion of insulin in the β-cells of the pancreas [54]. The rs2237892 polymorphism of *KCNQ1* was found to be associated with T2DM risk (Table 3), which is comparable to risk found in other ethnic groups in Asia [55].

ACE is involved in blood pressure regulation via catalysing the conversion of a vasoconstrictor in the RAS, which consequently influences insulin resistance [56]. The present study found the ACE insertion/deletion polymorphism to be associated with T2DM risk (Table 4). This finding aligns with a previous study of north Indians by Singh et al. [33], which found an increased risk associated with the D allele and the DD genotype. However, this association has been shown to vary between populations; although a 2010 meta-analysis [32] found an overall significant association, this did not remain significant in the west Asian sub-populations. 

*TCF7L2* promotes transcription of several proteins and is thought to be involved in the development of T2DM, primarily through influence on the pancreatic β-cells [57]. All three *TCF7L2* loci investigated in this study (rs12255372, rs7903146, and rs7901695) were found to be strongly associated with T2DM risk (Table 3). This agrees with the large body of research surrounding these polymorphisms, which finds *TCF7L2* to be one of the major polymorphisms contributing to T2DM risk, particularly in South Asia [35,58,59]. The effect size observed for these polymorphisms in Indian and South Asian populations makes this an ideal candidate gene for generating PRSs, however, varying OR values across India may mean PRSs would be population specific [60,61,62,63].

The binary logistic regression analyses confirmed the effects of the genotypes on T2DM risk. Inclusion of additional parameters into the regression model did not significantly change findings, suggesting the effects of these polymorphisms are independent (Tables S2 and S3). Regression analyses also confirmed that having a T2DM-affected parent can be an independent predictor of disease in the present study sample. A greater risk was associated with having a father affected with T2DM than a mother, and a further increased risk was associated with having both parents affected. These findings align with existing literature, which has observed high heritability of the disease [20]. Higher SBP was also found to increase T2DM risk, although with a relatively small effect size. This association is frequently observed in literature and is likely due to common pathways shared between hypertension and insulin resistance [56]. 

### 4.3. Polygenic Risk Score

The weighted PRS was found to be significantly higher in patients compared to the control group (Figure 1a) and was identified by the regression analysis as an independent predictor of T2DM risk (Table S3). Although the ROC curve analysis revealed that the weighted PRS alone was not a significantly more effective predictor of T2DM disease status than the clinical variables (Figure 1b), it is notable that the weighted PRS could bring about more utility in screening for T2DM. This is due to the ability to identify high-risk individuals at earlier ages before the onset of clinical parameters, such as high WHR or hypertension. In addition, variables such as dyslipidaemia can result from T2DM and so may be inappropriate for screening, as they are more likely to be present after the disease has already developed [44].

Weighted PRS in combination with the clinical variables was found to have the greatest discrimination power of the models tested (Figure 1b), although the utility of such a model may be context dependent. Lewis and Vassos [64] identified five points throughout the lifespan of which PRS could have a clinical application, such as risk prediction at birth and treatment decision-making. It is therefore important to consider clinical applications when determining which variables are appropriate to use in conjunction with the PRS. 

### 4.4. Limitations of the Study 

There are some limitations of our study which should be taken into consideration in interpretations and usage. The sample size of this study is limited; therefore, some expected associations were not detected at some loci (e.g., IGF2BP2, PPARG2). The findings from this study may have low generalisability due to the nature of the population studied. For example, the PRS developed is population specific and therefore may not be applicable to other populations due to variation in the risk associated with the polymorphisms, as well as complexities arising from gene–gene and gene–environment interactions [65].

## 5. Conclusions

This study observed the *GSTT1*(rs17856199), *GSTM1*(rs366631), *GSTP1*(rs1695), *KCNQ1*(rs2237892), *ACE*(rs4646994), and *TCF7L2*(rs12255372; rs7903146; rs7901695) loci to be associated with T2DM in a north Indian population of Jat Sikhs. No association was found with *IGF2BP2*(rs4402960) or *PPARG2*(rs1801282). One strength of the present study is the genetic isolation of the population due to the endogamous nature of Jat Sikhs. Increased linkage disequilibrium and potentially higher risk allele frequencies can result in increased statistical power to detect disease associations when compared to larger outbred populations. This study has some limitations, which include limited sample size from a specific population and analyses of only selected loci, which may limit the generalisation of these results. 

PRS was found to be a predictor of T2DM status, both independently and in combination with clinical variables. Further research is required to better establish these associations and to determine the clinical and public health utility of PRSs. As genetic association studies move towards analysing the combined effect of multiple polymorphisms, there is a need to better assess the predictive performance of PRSs, both independently and in combination with other risk factors, to determine clinical utility. Additionally, more investigation is required to establish how to apply these findings to clinical and public health settings in a practicable and ethical manner. 

## Figures and Tables

**Table 1:** Characteristic features of genes analysed in this study.

Gene	Role	Putative Pathological Function	rs Number	Association Study Findings
GSTT1 Glutathione S-transferase theta 1	Production of glutathione	Detoxification and inflammation	rs17856199	↑ risk (Nath et al., 2019) [21]
GSTM1 Glutathione S-transferase mu 1	Production of glutathione	As above	rs366631	↑ risk (Nath et al., 2019) [21]
GSTP1 Glutathione S-transferase pi 1	Production of glutathione	As above	rs1695	Consensus inconclusive (Saadat, 2017) [22]; ↑ risk in north India Mastana et al., 2013) [23]
KCNQ1 Potassium voltage-gated channel subfamily Q member 1	Voltage-gated potassium channel	Channels in pancreatic β-cells involved in regulating insulin secretion	rs2237892	↑ risk (Yu et al., 2020) [24]; inconsistent findings in India (Been et al., 2011; Phani et al., 2016a) [25,26]
IGF2BP2 Insulin-like growth factor 2 mRNA-binding protein 2	Regulator of IGF2 translation	IGF2 associated with reduced first-phase insulin secretion	rs4402960	↑ risk (Huang et al., 2017a; Rao et al., 2016) [27,28]
PPARG2 Peroxisome proliferator-activated receptor gamma 2	Nuclear receptor	Regulation of adipocyte differentiation and glucose and insulin sensitivity	rs1801282	↓ risk (Sarhangi et al., 2020; Majid et al., 2017) [29,30] some inconsistent findings (Phani et al., 2016b) [31]
ACE Angiotensin-converting enzyme	Angiotensin-converting enzyme	Angiotensin conversion affecting inflammation	rs4646994	↑ risk associated with D allele/DD genotype (Niu et al., 2010; Singh et al., 2006; Raza et al., 2017) [32,33,34]
TCF7L2 Transcription factor 7-like 2	Transcription factor	Apoptosis, proliferation, and functioning of pancreatic β-cells	rs12255372	↑ risk (Peng et al., 2013) [35]
TCF7L2	Transcription factor	As above	rs7903146	↑ risk (Peng et al., 2013) [35]
TCF7L2	Transcription factor	As above	rs7901695	↑ risk (Peng et al., 2013) [35]

Legend: ↑ increases risk. ↓ decreases risk.

**Table 2:** Genotype frequencies, allele frequencies, and HWE of 10 loci.

Gene/Position	Group	Total	GF (% Distribution)			MAF (±SE)	HWE *p* Value
			Null	Wild type			
*GSTT1*	Patients	225	70 (31)	155 (69)		N/A	N/A
rs17856199	Controls	231	40 (17)	191 (83)		N/A	N/A
			Null	Wild type			
*GSTM1*	Patients	225	119 (53)	106 (47)		N/A	N/A
rs366631	Controls	231	66 (29)	165 (71)		N/A	N/A
			I/I	I/V	V/V	V	
*GSTP1*	Patients	225	82 (36)	99 (44)	44 (20)	0.416 (±0.023)	0.158
rs1695	Controls	230	104 (45)	108 (47)	18 (8)	0.313 (±0.022)	0.164
			C/C	C/T	T/T	T	
*KCNQ1*	Patients	225	160 (71)	62 (28)	3 (1)	0.151 (±0.017)	0.267
rs2237892	Controls	230	137 (60)	86 (37)	7 (3)	0.217 (±0.019)	0.134
			G/G	G/T	T/T	T	
*IGF2BP2*	Patients	225	58 (26)	118 (52)	49 (22)	0.480 (±0.024)	0.448
rs4402960	Controls	230	58 (25)	119 (52)	53 (23)	0.489 (±0.023)	0.593
			P/P	P/A	A/A	A	
*PPARG2*	Patients	223	184 (83)	33 (15)	6 (3)	0.101 (±0.014)	0.006 *
rs1801282	Controls	230	178 (77)	48 (21)	4 (2)	0.122 (±0.015)	0.715
			I/I	I/D	D/D	I	
*ACE*	Patients	225	28 (12)	105 (47)	92 (41)	0.358 (±0.023)	0.816
rs4646994	Controls	231	60 (26)	123 (53)	48 (21)	0.526 (±0.023)	0.303
			G/G	G/T	T/T	T	
*TCF7L2*	Patients	223	127 (57)	85 (38)	11 (5)	0.240 (±0.020)	0.500
rs12255372	Controls	226	172 (76)	48 (21)	6 (3)	0.133 (±0.016)	0.244
			C/C	C/T	T/T	T	
*TCF7L2*	Patients	224	131 (58)	78 (35)	15 (7)	0.241 (±0.020)	0.469
rs7903146	Controls	230	151 (66)	74 (32)	5 (2)	0.183 (±0.018)	0.238
			C/C	C/T	T/T	C	
*TCF7L2*	Patients	225	38 (17)	106 (47)	81 (36)	0.404 (±0.023)	0.740
rs7901695	Controls	228	17 (7)	75 (33)	136 (60)	0.239 (±0.020)	0.148

Legend: GF = genotype frequency; MAF = minor allele frequency; HWE = Hardy–Weinberg equilibrium; * significance at *p*  <  0.05. Note: allele frequencies are not calculated for *GSTT1* or *GSTM1*, as the wild-type genotype cannot be separated into homozygotes and heterozygotes.

**Table 3:** Odds ratios under different models of association.

Gene/ Position	Model	Genotype/Allele	Crude OR (95% CI)	Crude *p* Value	Adjusted OR (95%CI) for Age and BMI	Adjusted *p* Value
*GSTT1*	Recessive	Wild type	1.00 (ref)		N/A	
rs17856199		Null	2.16 (1.39–3.36)	0.001 *	N/A	N/A
*GSTM1*	Recessive	Wild type	1.00 (ref)		N/A	
rs366631		Null	2.81 (1.91–4.13)	<0.001 *	N/A	N/A
*GSTP1*	Codominant	I/I	1.00 (ref)		1.00 (ref)	
rs1695		I/V	1.16 (0.78–1.73)	<0.001 **	1.16 (0.77–1.73)	0.0014 **
		V/V	3.10 (1.67–5.76)	<0.001 *	3.03 (1.61–5.67)	0.0014 *
	Dominant	I/I	1.00 (ref)		1.00 (ref)	
		I/V–V/V	1.44 (0.99–2.10)	0.057	1.42 (0.97–2.08)	0.069
	Recessive	I/I–I/V	1.00 (ref)		1.00 (ref)	
		V/V	2.86 (1.60–5.13)	<0.001 *	2.80 (1.55–5.05)	0.0004 *
	Log–additive	V	1.56 (1.18–2.05)	0.0014 *	1.54 (1.17–2.03)	0.0021 *
*KCNQ1*	Codominant	T/T	1.00 (ref)		1.00 (ref)	
rs2237892		C/T	1.68 (0.42–6.76)	0.026 **	1.76 (0.44–7.14)	0.033 **
		C/C	2.73 (0.69–10.74)	0.026 **	2.80 (0.71–11.13)	0.033 **
	Dominant	T/T	1.00 (ref)		1.00 (ref)	
		C/T–C/C	2.32 (0.59–9.10)	0.21	2.40 (0.61–9.47)	0.19
	Recessive	T/T–C/T	1.00 (ref)		1.00 (ref)	
		C/C	1.67 (1.13–2.47)	0.0096 *	1.65 (1.11–2.44)	0.013 *
	Log–additive	C	1.63 (1.14–2.33)	0.007 *	1.61 (1.12–2.31)	0.0091 *
*IGF2BP2*	Codominant	T/T	1.00 (ref)		1.00 (ref)	
rs4402960		G/T	1.07 (0.67–1.71)	0.95	0.99 (0.62–1.59)	0.99
		G/G	1.08 (0.64–1.84)	0.95	0.97 (0.56–1.67)	0.99
	Dominant	T/T	1.00 (ref)		1.00 (ref)	
		G/T–G/G	1.08 (0.69–1.67)	0.75	0.98 (0.63–1.54)	0.94
	Recessive	T/T–G/T	1.00 (ref)		1.00 (ref)	
		G/G	1.03 (0.68–1.57)	0.89	0.98 (0.64–1.50)	0.92
	Log–additive	G	1.04 (0.80–1.36)	0.78	0.99 (0.75–1.29)	0.91
*PPARG2*	Codominant	A/A	1.00 (ref)		1.00 (ref)	
rs1801282		P/A	0.46 (0.12–1.75)	0.20	0.44 (0.11–1.69)	0.18
		P/P	0.69 (0.19–2.48)	0.20	0.67 (0.19–2.46)	0.18
	Dominant	A/A	1.00 (ref)		1.00 (ref)	
		P/A–P/P	0.64 (0.18–2.30)	0.49	0.62 (0.17–2.26)	0.47
	Recessive	A/A–P/A	1.00 (ref)		1.00 (ref)	
		P/P	1.38 (0.87–2.19)	0.17	1.40 (0.88–2.24)	0.16
	Log–additive	P	1.21 (0.81–1.81)	0.34	1.22 (0.82–1.83)	0.32
*ACE*	Codominant	I/I	1.00 (ref)		1.00 (ref)	
rs4646994		I/D	1.83 (1.09–3.07)	<0.001 *	1.86 (1.10–3.14)	<0.0001 *
		D/D	4.11 (2.33–7.25)	<0.001 *	4.19 (2.36–7.44)	<0.0001 *
	Dominant	I/I	1.00 (ref)		1.00 (ref)	
		I/D–D/D	2.47 (1.51–4.04)	<0.001 *	2.52 (1.53–4.14)	<0.001 *
	Recessive	I/I–I/D	1.00 (ref)		1.00 (ref)	
		D/D	2.64 (1.74–3.99)	<0.001 *	2.66 (1.75–4.04)	<0.001 *
	Log–additive	D	2.06 (1.55–2.72)	<0.001 *	2.07 (1.56–2.75)	<0.001 *
*TCF7L2*	Codominant	G/G	1.00 (ref)		1.00 (ref)	
rs12255372		G/T	2.40 (1.57–3.66)	<0.001 *	2.42 (1.58–3.70)	<0.001 *
		T/T	2.48 (0.89–6.89)	<0.001 **	2.20 (0.78–6.19)	<0.001 **
	Dominant	G/G	1.00 (ref)		1.00 (ref)	
		G/T–T/T	2.41 (1.61–3.61)	<0.001 *	2.39 (1.59–3.60)	<0.001 *
	Recessive	G/G–G/T	1.00 (ref)		1.00 (ref)	
		T/T	1.90 (0.69–5.24)	0.20	1.67 (0.60–4.67)	0.32
	Log–additive	T	2.06 (1.45–2.93)	<0.001 *	2.03 (1.42–2.90)	<0.001 *
*TCF7L2*	Codominant	C/C	1.00 (ref)		1.00 (ref)	
rs7903146		C/T	1.21 (0.82–1.80)	0.036 **	1.23 (0.82–1.83)	0.031 **
		T/T	3.46 (1.22–9.77)	0.036 *	3.59 (1.26–10.22)	0.031 *
	Dominant	C/C	1.00 (ref)		1.00 (ref)	
		C/T–T/T	1.36 (0.93–1.99)	0.12	1.38 (0.94–2.02)	0.10
	Recessive	C/C–C/T	1.00 (ref)		1.00 (ref)	
		T/T	3.23 (1.15–9.04)	0.017 *	3.34 (1.18–9.44)	0.014 *
	Log–additive	T	1.43 (1.03–1.97)	0.03 *	1.45 (1.04–2.01)	0.026 *
*TCF7L2*	Codominant	T/T	1.00 (ref)		1.00 (ref)	
rs7901695		C/T	2.37 (1.58–3.55)	<0.001 *	2.47 (1.64–3.73)	<0.001 *
		C/C	3.75 (1.99–7.08)	<0.001 *	3.76 (1.98–7.14)	<0.001 *
	Dominant	T/T	1.00 (ref)		1.00 (ref)	
		C/T–C/C	2.63 (1.80–3.84)	<0.001 *	2.72 (1.85–3.99)	<0.001 *
	Recessive	T/T–C/T	1.00 (ref)		1.00 (ref)	
		C/C	2.52 (1.38–4.62)	0.0019 *	2.49 (1.35–4.57)	0.0024 *
	Log–additive	C	2.08 (1.56–2.77)	<0.001 *	2.11 (1.58–2.82)	<0.001 *

Legend: OR = odds ratio; * significance at *p*  <  0.05; ** significance at *p* < 0.05 but not 95% CI.

**Table 4:** Odds ratios for *TCF7L2* haplotypes.

Haplotype	Haplotype Frequency (%)		Crude OR (95% CI)	Crude *p* Value	Adjusted OR (95%CI) for Age and BMI	Adjusted *p* Value
	Controls	Patients				
GCT	104 (45.0)	127 (56.3)	1.00 (ref)	-	1.00 (ref)	-
GCC	47 (20.4)	39 (17.2)	2.36 (1.55–3.61)	<0.001 *	2.34 (1.53–3.57)	<0.001 *
GTT	26 (11.0)	21 (9.5)	2.64 (1.48–4.73)	0.001 *	2.62 (1.47–4.67)	0.001 *
TCT	19 (8.1)	14 (6.3)	2.69 (1.45–4.98)	0.002 *	2.63 (1.41–4.90)	0.002 *
TCC	12 (5.3)	4 (2.0)	8.02 (2.95–21.77)	<0.001 *	8.12 (2.98–22.09)	<0.001 *
GTC	11 (5.0)	8 (3.7)	2.39 (1.09–5.21)	0.029 *	2.61 (1.19–5.74)	0.018 *
TTT	9 (3.7)	9 (3.9)	1.36 (0.54–3.43)	0.510	1.29 (0.49–3.39)	0.600
TTC	3 (1.4)	2 (1.1)	4.75 (0.71–31.58)	0.110	4.85 (0.69–34.28)	0.110

Legend: *TCF7L2* SNP order: rs12255372, rs7903146, and rs7901695; OR = odds ratio; * significance at *p*  <  0.05.

## Data Availability

The data presented in this study are available on request from the corresponding author. Due to ethical, privacy and consent issues, individual data is not available in public database.

## Supplementary Materials

The following supporting information can be downloaded at: https://www.mdpi.com/article/10.3390/ijerph20043729/s1, Table S1: Anthropometric and clinical parameters of participants; Table S2: Binary logistic regression of different genetic loci with the inclusion of demographic, anthropometric, and clinical parameters; Table S3: Binary logistic regression of weighted PRS alone and with inclusion of demographic, anthropometric, and clinical parameters.
