# Supplementary material for: Genetic Risk Scores for the Determination of Type 2 Diabetes Mellitus (T2DM) in North India
Source: Int J Environ Res Public Health. 2023 Feb 20;20(4):3729. doi: 10.3390/ijerph20043729 (PMC9959290; doi:10.3390/ijerph20043729)
Supplement: Supplementary file 1 [file ijerph-20-03729-s001.zip › ijerph-2100487-supplementary.pdf]

Table S.1 – Anthropometric and clinical parameters of participants

**Characteristic**	**Group**	***N***	**Mean**	**SD**	**SE Mean**	***t*-test *P* value**
Age (years)	Patients	225	58.136	11.349	0.757	0.010*
	Controls	231	55.131	13.525	0.890	
Height (cm)	Patients	225	164.128	9.707	0.647	0.497
	Controls	231	163.523	9.303	0.612	
Weight (kg)	Patients	225	73.004	14.387	0.959	0.189
	Controls	231	71.317	12.983	0.854	
BMI	Patients	225	27.048	4.564	0.304	0.417
	Controls	231	26.701	4.557	0.300	
Waist (in.)	Patients	225	37.952	3.992	0.266	0.002*
	Controls	231	36.841	3.609	0.237	
Hip (in.)	Patients	225	39.121	3.631	0.242	0.647
	Controls	231	39.280	3.758	0.247	
WHR	Patients	225	0.970	0.055	0.004	<0.001*
	Controls	231	0.940	0.059	0.004	
Bodyfat (%)	Patients	225	35.586	9.810	0.654	0.911
	Controls	231	35.487	9.203	0.605	
SBP (mmHg)	Patients	225	148.213	23.118	1.541	<0.001*
	Controls	231	140.221	18.477	1.216	
DBP (mmHg)	Patients	225	85.049	12.206	0.814	0.054
	Controls	231	83.108	8.900	0.586	
Chol (mg/dL)	Patients	225	198.258	47.082	3.139	0.059
	Controls	231	207.087	52.163	3.432	
TG (mg/dL)	Patients	225	177.147	96.636	6.442	0.023*
	Controls	231	158.178	79.564	5.235	
HDL (mg/dL)	Patients	225	42.914	9.685	0.646	0.009*
	Controls	231	44.951	6.440	0.424	
LDL (mg/dL)	Patients	225	119.914	42.906	2.860	0.017*
	Controls	231	130.500	50.602	3.329	
VLDL (mg/dL)	Patients	225	35.429	19.327	1.288	0.023*
	Controls	231	31.636	15.913	1.047	

SD = standard deviation; SE = standard error; BMI = body mass index; WHR = waist-to-hip ratio; SBP = systolic blood pressure; DBP = diastolic blood pressure; Chol = cholesterol; TG = triglycerides; HDL = high-density lipoprotein; LDL = low-density lipoprotein; VLDL = very low-density lipoprotein

*Significance at $P < 0.05$

Table S.2 - Binary logistic regression of different genetic loci with the inclusion of demographic, anthropometric and clinical parameters

Predictor variable	β	Sig.	Exp(β)	95%CI for Exp(β)	
				Lower	Upper
GSTT1 (Null)	0.505	0.103	1.658	0.903	3.045
***GSTM1* (Null)**	**1.210**	**<0.001**	**3.355**	**1.953**	**5.763**
***GSTP1* rs1695**		**0.004**			
(I/V)	0.112	0.692	1.119	0.643	1.946
**(V/V)**	**1.433**	**0.001**	**4.193**	**1.750**	**10.048**
***KCNQ1* rs2237892**		**0.002**			
(C/T)	-0.650	0.510	0.522	0.075	3.611
(C/C)	0.397	0.678	1.488	0.228	9.691
IGF2BP2 rs4402960		0.888			
(G/T)	0.094	0.772	1.098	0.581	2.075
(G/G)	0.181	0.626	1.199	0.578	2.488
PPARG2 rs1801282		0.118			
(P/A)	-1.513	0.132	0.220	0.031	1.575
(P/P)	-0.853	0.366	0.426	0.067	2.709
***ACE* rs4646994**		**<0.001**			
(I/D)	0.585	0.098	1.795	0.898	3.589
**(D/D)**	**1.585**	**<0.001**	**4.880**	**2.280**	**10.447**
TCF7L2 rs12255372		0.052			
**(G/T)**	**0.673**	**0.023**	**1.960**	**1.096**	**3.507**
(T/T)	0.751	0.255	2.120	0.581	7.741
***TCF7L2* rs7903146**		**0.005**			
(C/T)	0.396	0.161	1.486	0.854	2.586
**(T/T)**	**2.166**	**0.002**	**8.725**	**2.239**	**34.011**
***TCF7L2* rs7901695**		**<0.001**			
**(C/T)**	**1.168**	**<0.001**	**3.215**	**1.857**	**5.566**
**(C/C)**	**1.426**	**0.001**	**4.163**	**1.795**	**9.658**
Sex (male)	0.416	0.867	1.517	0.012	196.560
Age (years)	0.015	0.532	1.015	0.968	1.066
**Parent affected**		**<0.001**			
**(Father affected)**	**1.502**	**0.002**	**4.490**	**1.771**	**11.388**
**(Mother affected)**	**1.246**	**0.009**	**3.478**	**1.371**	**8.824**
**(Both parents affected)**	**2.228**	**0.002**	**9.279**	**2.258**	**38.127**
BMI	0.036	0.448	1.037	0.945	1.138
Waist (in.)	0.215	0.275	1.240	0.842	1.825
**Hip (in.)**	**-0.242**	**0.001**	**0.785**	**0.681**	**0.905**
Bodyfat (%)	0.017	0.907	1.017	0.765	1.353
**SBP (mmHg)**	**0.024**	**0.002**	**1.024**	**1.009**	**1.040**
DBP (mmHg)	-0.021	0.145	0.979	0.952	1.007
**Cholesterol (mg/dL)**	**-0.008**	**0.008**	**0.992**	**0.986**	**0.998**

TG (mg/dL)	0.003	0.061	1.003	1.000	1.006
HDL (mg/dL)	-0.026	0.127	0.974	0.942	1.007
Constant	-2.848	0.345	0.058		

Table S.3 - Binary logistic regression of weighted PRS alone and with inclusion of demographic, anthropometric and clinical parameters

Predictor variable	β	Sig.	Exp(β)	95% CI for Exp(β)	
				Lower	Upper
		Regression of weighted PRS only			
**Weighted PRS**	**0.358**	**<0.001**	**1.430**	**1.327**	**1.541**
**Constant**	**-4.903**	**<0.001**	**0.007**		
		Regression of weighted PRS and covariates			
**Weighted PRS**	**0.349**	**<0.001**	**1.418**	**1.305**	**1.542**
Sex (male)	0.169	0.941	1.184	0.013	107.391
Age (years)	0.013	0.547	1.013	0.970	1.059
**Parent affected**		**<0.001**			
**(Father affected)**	**1.505**	**0.001**	**4.503**	**1.906**	**10.640**
**(Mother affected)**	**1.035**	**0.015**	**2.815**	**1.224**	**6.475**
**(Both parents affected)**	**2.452**	**0.001**	**11.607**	**2.836**	**47.509**
BMI	0.034	0.435	1.034	0.950	1.126
Waist (in.)	0.246	0.180	1.278	0.892	1.831
**Hip (in.)**	**-0.258**	**<0.001**	**0.773**	**0.675**	**0.885**
Bodyfat (%)	0.007	0.961	1.007	0.773	1.312
**SBP (mmHg)**	**0.020**	**0.005**	**1.020**	**1.006**	**1.035**
DBP (mmHg)	-0.018	0.183	0.982	0.957	1.008
**Cholesterol (mg/dL)**	**-0.006**	**0.023**	**0.994**	**0.988**	**0.999**
TG (mg/dL)	0.002	0.282	1.002	0.999	1.005
HDL (mg/dL)	-0.021	0.181	0.979	0.949	1.010
**Constant**	**-5.672**	**0.029**	**0.003**		

β = regression coefficient

Predictors significant at $P < 0.05$ indicated in bold
